# Nutritional and Bioactive Profiling of *Cucumis melo* L. By-Products: Towards a Circular Food Economy

**DOI:** 10.3390/molecules30061287

**Published:** 2025-03-13

**Authors:** Mafalda Alexandra Silva, Tânia Gonçalves Albuquerque, Diana Melo Ferreira, Rita C. Alves, Maria Beatriz P. P. Oliveira, Helena S. Costa

**Affiliations:** 1 Research and Development Unit, Department of Food and Nutrition, National Institute of Health Dr. Ricardo Jorge, Avenida Padre Cruz, 1649-016 Lisbon, Portugal; mafalda.silva@insa.min-saude.pt (M.A.S.); helena.costa@insa.min-saude.pt (H.S.C.); 2 REQUIMTE/LAQV, Department of Chemical Sciences, Faculty of Pharmacy, University of Porto, 4050-313 Porto, Portugal; melo_dian@hotmail.com (D.M.F.); rita.c.alves@gmail.com (R.C.A.); beatoliv@ff.up.pt (M.B.P.P.O.)

**Keywords:** melon, nutritional composition, antioxidant potential, functional foods, sustainability, peel and seeds, food waste

## Abstract

Food waste, due to the high quantities produced, becomes a significant environmental, economic, and social challenge worldwide. Simultaneously, the rising prevalence of chronic diseases has intensified the demand for healthier food options. A promising approach to address these issues involves the valorisation of food by-products for the development of innovative and healthier food products. *Cucumis melo* L., commonly consumed as a fruit, generates peels and seeds that are typically discarded. In the present study, the nutritional composition and antioxidant potential of pulp, peel, and seeds of *C. melo* L. (yellow and green melon) were comprehensively evaluated. The seeds were identified as a rich source of dietary fibre (39.0 and 39.7 g/100 g dw; *p* > 0.05) and protein (21.0 and 21.3 g/100 g dw; *p* > 0.05), exhibiting an appealing fatty acid profile. The peel contains high levels of dietary fibre (39.7 and 47.1 g/100 g dw; *p* > 0.05) and total phenolic compounds (1976 and 2212 mg GAE/100 g dw; *p* > 0.05), suggesting significant bioactive potential. The peels showed a high antioxidant capacity for both methods used, DPPH• (120 and 144 mg TE/100 g dw; *p* > 0.05) and FRAP (6146 and 7408 mg TE/100 g dw; *p* > 0.05) assays. Potassium emerged as the predominant mineral in the seeds (799 and 805 mg/100 dw; *p* > 0.05), while glutamic acid was the most abundant amino acid (4161 and 4327 mg/100 g dw; *p* > 0.05). These findings emphasise the antioxidant and nutritional properties of *C. melo* L. by-products, highlighting their potential for inclusion in novel food formulations. This study not only advances the understanding of *C. melo* L. properties but also supports the reduction of food waste and promotes sustainability within the food supply chain.

## 1. Introduction

According to the data from FAO, the European Union generates approximately 88 million tonnes of food waste annually, with associated costs of around 143 billion euros [1]. Food waste is not only a social issue but also has a significant negative impact on the economic and environmental sectors, with poor management leading to severe consequences for human health. Reducing food waste aligns with the European Commission’s goals for sustainable development, supports the fight against climate change, allows households, companies, and farmers to save money, and contributes to the eradication of hunger and malnutrition that affects millions of people around the world [1,2]. One promising approach to tackling food waste involves the utilisation of by-products from the food industry, particularly the fruit sector, where a significant amount of by-products is discarded. These by-products can be used as valuable sources of functional ingredients with health-promoting properties [3,4,5,6,7,8,9].

The world’s population is expected to reach 9.8 billion by 2050, presenting enormous challenges in ensuring equitable access to safe, nutritious, and healthy food for all. Reducing food waste could play a critical role in achieving food security and sustainability to meet these challenges [10].

Fruits and vegetables are essential in the human diet, providing vital nutrients like dietary fibre, vitamins, minerals, and bioactive phytochemicals. Global fruit production reached 933 million tonnes in 2022. However, this sector is among the largest contributors to food waste, primarily due to the perishability of these products, which often leads to their disposal as they fail to meet quality standards [1]. Thus, the food waste resulting from the production of fruits and vegetables, in particular peels, seeds, and pulp, could serve as raw materials for extracting bioactive compounds or for enriching food products [11,12,13,14].

*Cucumis melo* L., commonly known as melon, is a widely cultivated fruit belonging to the Cucurbitaceae family. Its diverse fruit morphology contributes to its popularity [15,16]. Since it is a widely consumed fruit, it generates large amounts of peels and seeds that are discarded until now [17]. Data from FAOSTAT showed that the worldwide production of melon was approximately 29 million tonnes in 2023. Since its by-products represent about 28–51% of the total melon weight, about 8–15 million tonnes of by-products are generated [18,19]. The nutritional value and possible uses of melon by-products in the food industries have been studied due to their demonstrated benefits. While research has traditionally focused on the consumption of melon pulp, recent studies have highlighted the relevance of fruit processing by-products as valuable resources. A recent study developed a value-added drink with peel and seed extracts with a long shelf life in terms of the stability of bioactive compounds and with good sensory scores [20]. On the other hand, the fatty acid profile and high protein content of melon seeds made it possible to develop a butter made from melon seeds, rich in protein that can be applied in a wide range of bakery products [21]. Additionally, melon seed oil, along with pumpkin seed oil, has been used as pork fat substitutes in traditional burgers, reducing the saturated fat content and increasing the polyunsaturated fatty acids proportion [22]. In another study, defatted melon seed residue was used to develop a bread considered a source of fibre, and with enhanced protein and lipid content, improving bread nutritional quality [23]. In addition to their direct application in food, melon by-products have also been explored for their ability to provide high-value bioactive compounds. These compounds, such as phenolic compounds, have shown beneficial properties for health. In this sense, a recent study demonstrated that melon seed extracts have potent antidiabetic as well as antioxidant activity along with a hypolipidemic effect [24]. On the other hand, melon rinds have also been shown to have antioxidant and prebiotic potential, which could improve human gut health [25]. Moreover, the results obtained by Rolim et al. (2018) suggest that extracts from melon by-products, in addition to presenting high antioxidant activity, also have effective biological activity against the growth of human tumour cells [26].

Despite these advancements, to the best of our knowledge, a significant gap in the literature remains regarding the detailed nutritional characterisation and functionality of melon peel, as well as its potential applications. To address this gap, our study provides a comprehensive nutritional assessment of melon peel while also exploring its functional properties and industrial applicability. Furthermore, unlike previous studies that have focused separately on melon peel or seeds, our approach considers the by-product as a whole. Thus, our goal is to maximise the full potential of melon by-products by developing strategies that facilitate their complete utilisation. In line with this, we also employed sample preparation techniques that enhance industrial scalability, choosing dehydration over freeze-drying to ensure feasibility in large-scale food applications.

Therefore, this study aims to provide a comprehensive characterisation of the nutritional composition, bioactive properties, and potential health benefits of the pulp, peel, and seeds of *C. melo* L. (green and yellow melons). By exploring these underutilised by-products, this study will contribute to their valorisation as functional food ingredients, offering solutions to food waste reduction and promoting sustainability in the food supply chain.

## 2. Results and Discussion

### 2.1. Proximate Composition

The nutritional composition of the pulp, peel, and seeds of *Cucumis melo* L. highlights their potential as functional food ingredients (Table 1). While melon pulp primarily consists of available carbohydrates (68.2 and 69.7 g/100 g dw for green and yellow melon, respectively), the peel and seeds offer a richer profile of vital nutrients and bioactive compounds that could address key dietary needs.

The protein content of melon seeds (21.0 and 21.3 g/100 g dw, for yellow and green melon, respectively) underscores their potential as a plant-based protein source. In the present study, the green melon had higher protein levels in both the pulp and peel compared to the yellow melon (*p* < 0.05). In the literature, Morais et al. (2017) [6] reported a higher peel protein content than those found in the present study, 15.1 g/100 g dw. For seeds, Raji & Orelaja et al. (2014) [27] reported very similar levels to those in the present study, 21.05 g/100 g. On the other hand, Morais et al. (2017) [6] reported lower contents, 15.6 g/100 g, for melon seeds. Proteins play an essential role in muscle maintenance, immune function, and metabolic processes, making these by-products valuable in developing high-protein foods or supplements. Although the peel and pulp showed lower protein levels, their contribution is still meaningful, particularly when considering their integration into composite food.

Melon seeds exhibited a significant fat content (green: 27.5; yellow: 28.5 g/100 g dw), aligning with previous findings in the literature [6,27,28]. This composition positions melon seeds as candidates for extraction of healthy oils or as ingredients in formulations targeting functional lipid enrichment. Some studies highlight the nutritional composition and natural bioactive and antioxidant compounds present in melon seed oil, which justify its biological properties and potential benefits for human health [29].

Dietary fibre is one of the most notable attributes of melon by-products, with seeds containing 39.7 and 39.0 g/100 g and the peel presenting even higher levels (39.7 and 47.1 g/100 g dw, for the green and yellow melon, respectively). These values exceed those reported in prior studies, further emphasising their potential [28,30]. Dietary fibre is crucial for gastrointestinal health, glycaemic control, and cholesterol reduction, making these by-products valuable for developing fibre-enriched functional foods such as bakery items, snacks, or supplements targeting digestive health.

Melon by-products demonstrate superior nutritional and functional properties, particularly in terms of dietary fibre and protein, compared to the pulp. These findings underscore their relevance as ingredients in developing functional foods to improve diet quality and address specific health concerns [31,32].

### 2.2. Fatty Acids Composition

The predominance of polyunsaturated fatty acids, particularly omega-6, coupled with the presence of monounsaturated fatty acids, highlights the nutritional and functional value of melon seeds. Their inclusion in food formulations or as an ingredient in health-focused products could contribute to the dietary intake of essential fatty acids and promote cardiovascular health [33].

The results for the fatty acid composition of *C. melo* L. seeds are presented in Table 2. The analysed melon seeds have a similar fatty acid profile, with a high proportion of polyunsaturated fatty acids, accounting for 62% and 67% of the total fatty acids in green and yellow melon seeds, mostly omega-6. Among these, linoleic acid emerged as the major component, with yellow melon seeds containing a higher concentration (18.5 g/100 g dw) than green melon seeds (16.1 g/100 g dw). This high linoleic acid content underscores the potential of melon seeds as a source of essential fatty acids, which have been associated with reduced risks of cardiovascular diseases [34].

In addition to linoleic acid, other notable fatty acids include oleic, palmitic, and stearic acids. Green melon seeds exhibited higher levels of oleic acid (6.05 g/100 g dw) and palmitic acid (2.48 g/100 g dw) than yellow melon seeds (4.47 g/100 g dw and 2.45 g/100 g dw, respectively). Conversely, yellow melon seeds had a higher content of stearic acid (1.43 g/100 g dw). These findings are consistent with previous reports, which also identified linoleic, oleic, palmitic, and stearic acids as the primary fatty acids in melon seeds [28,35].

The results align closely with those from the study by Mallek-Ayadi et al. (2019) [36], which reported a fatty acid distribution of 15% saturated fatty acids, 16% monounsaturated fatty acids, and 69% polyunsaturated fatty acids for the Maazoun variety of melon seeds. Differences in fatty acid composition between studies may stem from variables such as climatic conditions, soil characteristics, and the specific melon varieties analysed.

### 2.3. Amino Acid Composition

The amino acid profile of *C. melo* L. seeds supports its application in food systems as a plant-based protein source. Their high glutamic acid content could enhance flavour profiles through the natural umami taste, while arginine could provide additional cardiovascular benefits [37]. Despite some limitations in lysine content, the inclusion of these by-products in protein blends or fortified food products could help achieve a more balanced amino acid profile, serving diverse dietary needs.

The amino acid profile of *C. melo* L. seeds underscores their potential as a valuable source of dietary protein, particularly in the context of functional food development. As shown in Table 3, the seeds are rich in essential and non-essential amino acids, with notable differences between green and yellow melons.

The primary essential amino acids identified in the seeds were leucine and phenylalanine, which play critical roles in muscle protein synthesis, among other vital functions. The seeds contain all essential amino acids, contributing 19% and 24% of the total amino acid content for green and yellow melon seeds, respectively. The amino acid score, calculated according to FAO guidelines (2013) [38], revealed some deficiencies in specific amino acids (Table 4). Lysine was identified as the first limiting amino acid in both seed types of melon, which is common in plant-based proteins. However, yellow melon seeds exhibited slightly better amino acid scores than green melon seeds, suggesting their potential for inclusion in balanced protein formulations [39].

Among non-essential amino acids, glutamic acid, aspartic acid, arginine, and glycine were predominant, comprising 63% of the total amino acid content in green melon seeds and 58% in yellow melon seeds. Green melon seeds contained higher levels of glutamic (4327 mg/100 g dw) and aspartic acids (1896 mg/100 g dw), while yellow melon seeds were richer in arginine (1799 mg/100 g dw), leucine (1034 mg/100 g dw), glycine (1026 mg/100 g dw), and phenylalanine (706 mg/100 g dw). Cysteine was not detected in the analysed samples, which is consistent with findings by Hu & Ao (2007) [40] for “ChunLi” hybrid seeds.

The results obtained in this study are in agreement with several authors, who have consistently highlighted the robust amino acid profiles of melon seeds [39,40,41]. Hu & Ao (2007) [40] and Melo et al. (2000) [42] reported glutamic acid (17.5 and 19.5 g/100 g protein), arginine (15.6 and 12.2 g/100 g protein), and aspartic acid (9.65 and 9.07 g/100 g protein) as the three amino acids with the highest content for melon hybrid ‘ChunLi’ and AF-522 seeds, respectively. On the other hand, Mallek-Ayadi et al. (2019) [41], in addition to glutamic acid (20.5 g/100 g protein) and arginine (13 g/100 g protein), reported tryptophan (13 g/100 g protein) as the third amino acid with the highest content in melon seeds of the Maazoun cultivar.

### 2.4. Mineral Composition

The mineral profile of the analysed *C. melo* L. seeds reveals their potential as a rich source of essential nutrients, which could be advantageous in the development of functional food ingredients (Table 5). The main macrominerals identified in the melon seeds were K, P, and Mg. K was the most abundant one, with green melon seeds exhibiting the highest levels (805 mg/100 g dw), underscoring their potential role in maintaining fluid balance and supporting cardiovascular health. P and Mg were also found in notable amounts, with yellow melon seeds showing higher concentrations of both minerals (669 mg/100 g dw and 299 mg/100 g dw, respectively). These nutrients are essential for bone health, energy metabolism, and muscle function.

These results are in agreement with several authors, who reported that melon seeds are a valuable source of minerals [6,30,43]. While K levels reported in the current study were slightly lower than those in some earlier studies (Azhari et al. [43]: 9548 mg/100 g; Morais et al. [6]: 1887 mg/100 g; Mallek-Ayadi et al. [30]: 1149 mg/100 g), the Mg and P contents observed here are comparatively higher. These differences may reflect variations in cultivation conditions, such as soil type, climate, and melon variety.

In addition to the macro-minerals, the seeds also provide beneficial trace elements like Fe, Mn, and Zn. These microminerals are crucial for immune function, antioxidant protection, and enzymatic activity. Interestingly, the levels found in this study were higher than those reported in previous research [30], highlighting the nutritional value of these seeds herein studied.

The rich mineral profile of *C. melo* L. seeds supports their inclusion as functional ingredients in food formulations. K and Mg are particularly relevant in products targeting cardiovascular health, while P contributes to the formulation of energy-rich foods. The presence of trace minerals like Fe and Zn further enhances their potential for addressing micronutrient deficiencies in vulnerable populations [44,45].

### 2.5. Vitamin E

Vitamin E is a fat-soluble antioxidant known for its critical role in modulating inflammatory responses, enhancing immune function, and reducing the risk of chronic diseases, such as cardiovascular diseases, cancer, and neurodegenerative disorders like Alzheimer’s disease [46]. The vitamin E profile of *C. melo* L. by-products reveals their potential as valuable dietary sources of this essential nutrient (Table 6).

Among the by-products, yellow melon seeds had the highest vitamin E content (15 mg/100 g dw), while green melon peels showed a higher vitamin E content (20 mg/100 g dw) than the yellow melon peels (5.2 mg/100 g dw). In comparison, the pulp had relatively lower vitamin E concentrations, highlighting the by-products as a valuable source.

α-Tocopherol was the main component of vitamin E in the pulp, while the γ- tocopherol was the main vitamer present in the peels and seeds. The contents of γ-tocopherol in the seeds were 5.8 and 12 mg/100 g dw, while the peel had 13 and 2.9 mg/100 g dw for green and yellow melons, respectively. On the other hand, the α-tocopherol contents present in the seeds were 1.6 and 2.2 mg/100 g dw, while the peels had 4 and 1.3 mg/100 g dw for green and yellow melons, correspondingly. β-Tocopherol was not detected in the melon seeds. γ-Tocotrienol was the only tocotrienol present in all analysed samples. Rabadán et al., (2020) also found that γ-tocopherol was the main vitamer present in the seeds, with contents ranging from 99.81 to 456.73 mg/kg [47].

Melon peels contained higher vitamin E than pulp and seeds, probably due to their protective role against environmental stressors such as UV radiation, oxidation, and microbial attack [48]. Although the seeds have significantly higher fat contents, their metabolic focus is energy storage, not defence, so their vitamin E concentration may remain lower, as seeds prioritise lipid reserves for germination (triacylglycerols) rather than antioxidant protection. This distribution pattern aligns with findings in other fruits, such as papaya [49], where the peel also contained more vitamin E than the seeds. These results suggest that the biochemical composition of melon peel is optimised for defence against environmental stressors, while the pulp serves as a nutrient and water reservoir, and seeds function as energy storage units for future germination.

The high levels of γ-tocopherol in melon seeds and peels position these by-products as valuable sources of potent antioxidants. γ-Tocopherol has been shown to exhibit unique anti-inflammatory properties and may complement the antioxidant activity of α-tocopherol in reducing oxidative stress and inflammation [50]. The presence of γ-tocotrienol further enhances the potential of these by-products, given its emerging role in protecting against oxidative damage and supporting skin and cardiovascular health [51]. Incorporating *C. melo* L. by-products, particularly seeds and peels, into functional food formulations could increase the antioxidant capacity of these products while addressing consumer demand for natural, nutrient-rich ingredients.

### 2.6. Total Vitamin C

Vitamin C is a water-soluble antioxidant that plays an essential role in human health. It is involved in various metabolic processes, including the synthesis of amino acids (e.g., tyrosine and tryptophan), folic acid metabolism, and cholesterol reduction. Additionally, it supports the formation and maintenance of collagen, a key structural protein in connective tissues [52].

The vitamin C content of *C. melo* L. pulp and peels is presented in Table 7. No detectable levels of vitamin C were found in the seeds of either melon variety, likely due to the low water-soluble vitamin content in this part of the fruit. The green melon’s pulp and peel had higher levels of total vitamin C, with the peel (70.3 mg/100 g dw) exhibiting higher concentrations than the pulp (56.7 mg/100 g dw). Similarly, the peel of yellow melon contained more vitamin C (31.2 mg/100 g dw) than the pulp (26.2 mg/100 g dw), although overall levels were lower compared to the green melon. These variations can be attributed to several biochemical, genetic, or environmental factors, such as growing conditions, soil profile, and exposure to sunlight [53]. Şelale et al. (2011) analysed 42 melon pulps from different lines, and the vitamin C content ranged from 48.4 to 178.3 mg/kg, being higher than the values obtained in our study [54]. Across varieties, the peels consistently demonstrated higher vitamin C content than the pulps, highlighting their potential as a concentrated source of this essential nutrient. The peel is the outermost layer of the fruit and is directly exposed to various environmental factors. This exposure requires a higher concentration of antioxidants such as vitamin C to protect the fruit, leading to higher levels in the peel than the inner parts such as seeds and pulp [48,55].

Incorporating melon peels into food products or dietary supplements could provide a natural and sustainable source of vitamin C, meeting consumer demands for health-enhancing ingredients. These findings further support the valorisation of *C. melo* L. by-products as functional food components, reducing waste while delivering nutritional and health benefits [56].

### 2.7. Antioxidant Activity

Two different methods were used to determine the antioxidant activity: 2,2-diphenyl-1-picrylhydrazyl (DPPH•) and ferric-reducing antioxidant power (FRAP) assays.

The DPPH assay is a standard, simple, inexpensive, and rapid colorimetric method, as the radical is stable and does not need to be generated compared to the 2,2′-azino-bis(3-ethylbenzothiazoline-6-sulfonic acid) assay (ABTS). It can be used for thermally unstable compounds since radical elimination is measured at room temperature. However, DPPH radical chromogens are dissolved only in organic solvents. On the other hand, the ABTS cationic radical is soluble in organic and aqueous media. This assay can be used to screen both lipophilic and hydrophilic samples, similar to the cupric reducing antioxidant capacity (CUPRAC) method. FRAP is a simple method, with high reproducibility and simple instrumentation, which can be used on a wide range of samples. However, it can only be performed under acidic conditions. Background colour in the food matrix can be a problem and trigger absorbance modifications. These adverse effects could be more significant in discolouration reactions (ABTS and DPPH) than in colour-formation reactions (FRAP, CUPRAC) [57,58].

The results obtained for the pulp, peel, and seeds of *C. melo* L. are shown in Table 7. Melon peels presented a higher antioxidant capacity compared to the pulps and seeds analysed. The sample that presented a higher antioxidant activity through the DPPH• method was the green melon peel, 144 mg TE/100 g dw. Considering the analysed peels and pulps, the green melon had a higher antioxidant activity than the yellow one. In fact, green melon also has more phenolic compounds and vitamin C. Since these compounds have antioxidant properties, they are the main contributors to the higher antioxidant activity of green melon compared to yellow melon. In the case of seeds, the opposite was found (4.59 and 5.57 mg TE/100 g dw, for green and yellow melons, respectively). Morais et al. (2015) [59] analysed the melon pulp, peel, and seeds and obtained a higher antioxidant capacity for the melon peel, IC50 458.6 µg/mL, through the DPPH• assay, which is in agreement with the results obtained in the current study. The results obtained for the FRAP assay are in accordance with those obtained for the DPPH• method. The peel showed the highest antioxidant activity, 7408 and 6146 mg TE/100 g dw, for green and yellow melon, respectively. The peels showed a 3-fold higher antioxidant activity than the pulps. On the other hand, the green melon had higher antioxidant activity than the yellow melon. Morais et al. (2015) [59] obtained a higher antioxidant capacity, through the FRAP assay, for the melon peel, 49.5 µmol FeSO_4_/g dw, compared to the pulp (28.6 µmol FeSO_4_/g dw) and seeds (6.50 µmol FeSO_4_/g dw) analysed, results that are also in agreement with our study. Fruit peels generally exhibit higher antioxidant activity than seeds and pulps due to their richer content of phenolic compounds, flavonoids, vitamins C and E, and other bioactive compounds. These components are crucial in enhancing the antioxidant capacity of peels, making them valuable for potential health benefits [48,55].

Antioxidants are compounds that can protect against and stabilise free radical damage, which plays an important role in the development of many chronic diseases, such as cardiovascular disease, ageing, heart disease, and cancer, among others [60]. Thus, incorporating these by-products into food products could increase their antioxidant capacity and the dietary intake of these compounds [60].

### 2.8. Total Phenolics Compounds

Phenolic compounds are well known for their biological activities and therefore have several potential health benefits through their antioxidant, anti-inflammatory, anti-ageing, and antiproliferative properties [61].

Table 7 also shows the results obtained for the total phenolic content of the melon pulp and by-products. In general, the green melon presented a higher content of total phenolic compounds than the yellow melon. The peels showed the highest concentration of phenolic compounds, with 2212 and 1976 mg GAE/100 g dw for the green and yellow melon, respectively. The main classes of phenolic compounds identified in melon peels are phenolic acids, flavones, phenyl ethanoids, and phenolic alcohols [61], as well as hydroxybenzoic acids and flavonoids [30]. When comparing the pulp with the peel, the latter contains 2-fold (green melon) and 3-fold (yellow melon) the phenolic compounds content of the former. The difference between peel and seeds is even more pronounced: the peel had 11-fold (green melon) and 10-fold (yellow melon) higher total phenolic contents compared to the seeds. These results are consistent with other studies. For example, Morais et al. (2015) [59] reported higher levels of phenolic compounds in the peel (358 mg GAE/100 g dw) than in the seeds (100 mg GAE/100 g dw) and pulp (313 mg GAE/100 g dw). Fruit peels generally have a higher content of phenolic compounds compared to seeds and pulps due to their role in protecting the fruit and exposure to environmental stressors. Since the peel is the outermost layer of the fruit, it naturally accumulates more phenolics to serve as a protective barrier [48].

### 2.9. Sustainable Utilization of Melon By-Products

The results obtained in this study for the nutritional composition and antioxidant potential of melon by-products, seeds, and peel highlight their potential for inclusion in novel food formulations. The use of these by-products is aligned with the principles of the circular economy, which focuses on reintegrating materials that would otherwise be discarded back into the production cycle, thereby minimising waste and resource loss [62]. This approach not only reduces environmental impact but also fosters innovation by encouraging further research into new benefits and applications for these by-products. At the same time, consumer demand for natural, health-promoting ingredients has increased, driving the development of new products that incorporate these by-products. Melon peels and seeds contain significant levels of protein, dietary fibre, and bioactive compounds, such as antioxidants and vitamins, emphasising their potential as nutrient-rich ingredients. Their exploitation holds promise for innovation in the food industry and contributes to sustainable development. Moreover, the biological activities of the bioactive compounds present in these by-products can promote health and help prevent diseases, aligning with consumer preferences for healthier food options. In this context, valorising melon by-products can significantly contribute to the circular economy by reducing waste and promoting a more efficient use of natural resources. Their incorporation into the formulation, such as flours, functional beverages, nutritional supplements, and/or food additives, not only minimises environmental impacts but also enhances the value of food products. This fosters innovation and supports sustainable development goals, particularly those related to food security, health safety, and environmental protection.

## 3. Materials and Methods

### 3.1. Standards and Reagents

All chemicals and reagents were purchased from various commercial sources and were of analytical grade. Ultrapure and deionised water from the Milli-Q system (Millipore, Bedford, MA, USA) was used. L-Ascorbic acid, gallic acid, and Trolox (6-hydroxy-2,5,7,8-tetramethylchroman-2-carboxylic acid) were purchased from Sigma-Aldrich (Darmstadt, Germany). Food test materials were obtained from the Food Analysis Performance Assessment Scheme (FAPAS^®^) proficiency tests 2496 and 3244. Fatty acid methyl esters (FAMEs) standards were purchased from Supelco^®^ (Supelco^®^ 37 FAME Mix C4:0–C24:0 and Linoleic acid cis/trans isomers, Supelco, Bellefonte, PA, USA). Tocopherols (α, β, γ, and δ) and tocotrienols (α, β, γ, and δ) were obtained from Calbiochem (La Jolla, CA, USA). The internal standard tocol (2-methyl-2-(4,8,12-trimethyltridecyl)chroman-6-ol) was obtained from Matreya Inc. (State College, PA, USA). AccQ-Tag Chemistry kits were obtained from Waters Corporation Company (Milford, CT, USA). The working solutions were prepared by diluting the standard stock solution (Waters Amino Acid Hydrolysate standard) containing 2.5 mM of each amino acid (histidine (His), serine (Ser), arginine (Arg), glycine (Gly), aspartic acid (Asp), glutamic acid (Glu), threonine (Thr), alanine (Ala), proline (Pro), cysteine (Cys), lysine (Lys), tyrosine (Tyr), methionine (Met), valine (Val), isoleucine (Ile), leucine (Leu), and phenylalanine (Phe)). Working solutions were prepared from mono-element high-purity inductively coupled plasma stock standards containing 1000 mg/L of each element (calcium (Ca), magnesium (Mg), sodium (Na), potassium (K), phosphorus (P), iron (Fe), zinc (Zn), and manganese (Mn)).

### 3.2. Samples

*C. melo* L. samples were kindly supplied by two companies (Frutas A. R. Santos in Torres Vedras, Portugal, and Planície Verde in Rio Maior, Portugal) in 2021. Samples were distinguished between green melons (Grand Prix, Waikiki, D. Quixote, Pele de Sapo varieties) and yellow melons (Soleares variety). Then, they were manually divided between edible portions (pulp) and non-edible portions (peel and seeds). The pulp and peel were homogenised in a blender (Grindomix GM200, Retsch, Haan, Germany) at 5000 rpm for 1 min. The seeds were dried in an oven and ground using a domestic blender (Taurus, Barcelona, Spain). After homogenisation, the samples were stored under vacuum at −80 °C in the dark to avoid deterioration until analyses. For vitamin C determination, a stabilising solution (perchloric acid 10% (*v*/*v*) and 1% (*w*/*v*) metaphosphoric acid in ultrapure water) was added prior to storage.

### 3.3. Proximate Analysis

The different parts (pulp, peel, and seeds) of *C. melo* L. were analysed regarding their content of moisture, ash, total protein, total fat, and total dietary fibre. The available carbohydrates and energy values were obtained by calculation [63,64]. For quality assurance purposes, the test material FAPAS^®^ 2496 was analysed with these methods and compared with the assigned values.

To determine the moisture content, a dry air oven (Thermo Scientific Heraeus, Waltham, MA, USA) was used, following the official method of AOAC 931.04, 2023 [65]. For the ash content, samples were directly incinerated in a muffle furnace M 110 (500–550 °C) (Memmert GmbH, Schwabach, Germany), in accordance with the gravimetric method [65]. The Kjeldahl method was used to determine the protein content of the samples, using the block digestion system Foss Tecator 2006 Digestor and a Foss 2200 Kjeltec Auto Distillation unit (Foss, Hilleroed, Denmark) [65]. The Soxhlet extraction method (Soxtec™ 2050, Foss, Hilleroed, Denmark) with acid hydrolysis was used to obtain the total fat content, using petroleum ether as an extraction solvent. An enzymatic-gravimetric method was used to determine the total dietary fibre content of the samples, according to AOAC 985.29, 2023 [65].

### 3.4. Fatty Acids Analysis

The determination of the fatty acids profile was performed through a fat extraction with petroleum ether [66]. Subsequently, a cold transesterification was carried out with a methanolic solution of potassium hydroxide (2 M) and n-heptane according to the method described by Albuquerque et al. (2016) [66]. For analysis by gas chromatography coupled with flame ionisation detection, a SP-2560 capillary column (100 m × 0.25 mm i.d.; 0.2 μm film thickness, Supelco^®^, Bellefonte, PA, USA) was used. Helium was used as a carrier gas. A total of 1 μL of each sample to be analysed was injected in 50:1 split mode. Injector and detector temperatures were maintained at 240 °C. The following temperature ramp was used: a 60 °C (1 min) increase to 168 °C at 17 °C/min (hold 28 min) and then an increase to 235 °C at 4 °C/min (hold 15 min). To identify the FAMEs, a comparison of the retention times of the peaks in the sample was made with those of the pure standards Supelco^®^ 37 FAME Mix C4:0-C24:0 and cis/trans isomers of linoleic acid (Supelco^®^, Bellefonte, PA, USA).

The conversion of FAMEs into their fatty acids was carried out using the appropriate conversion factors, according to AOAC 996.06 [65]. The quantification of fatty acids was carried out by comparing the areas of their peaks with the peaks of the corresponding standard fatty acids, with each fatty acid being expressed as a percentage of total quantified fatty acids.

### 3.5. Amino Acid Analysis

Derivatisation and chromatographic separation were carried out for the amino acid analysis using the procedure described by Mota et al. (2016) [67]. Briefly, 25 mg of each sample was weighed into quartz digestion vials. Then, 200 µL of an internal standard solution (25 mM D-Norvaline) and 1 mL of hydrochloric acid (6 N) with 0.5% phenol were added. A closed-system microwave digestion equipment (Milestone ETHOS 1 Series, Milestone, Sorisole, Italy) was used to digest the samples. Afterwards, 1 mL of sodium hydroxide (6 N) was added to neutralise the extracts following hydrolysis. Ultrapure water was added to the extracts to achieve a final volume of 10 mL and then filtered. In order to perform the derivatisation procedure (55 °C, 10 min), 80 µL of borate buffer, 20 µL of derivatisation reagent (6-aminoquinolyl-N-hydroxysuccinimidyl carbamate), and 10 µL of sample were mixed in a chromatography vial.

To ensure quality, the test material FAPAS^®^ 3244 was analysed and compared with the assigned values.

A Waters™ Acquity UPLC system (Waters Corporation Company, Milford, MA, USA) with a photodiode array detector (PDA) and an Acquity UPLC BEH C18 column (100 mm × 2.1 mm, 1.7 µm) was used for separation and quantification. A flow rate of 0.7 mL/min was used, and the column temperature was set at 55 °C. Two eluents—AccQTag ultra eluent A diluted in 95% ultrapure water and AccQTag ultra eluent B—from Waters Corporation Company constituted the mobile phase. The elution gradient was set as follows: 0–0.54 min, 99.9% A–0.1% B; 5.74 min, 90.9% A–9.1% B; 7.74 min, 78.8% A–21.2% B; 8.04 min, 40.4% A–59.6% B; 8.70–10 min, 99.9% A–0.1% B. The injection volume was 1 µL, and the samples were monitored at 260 nm. The Empower™ software version 2.0 (Waters, Milford, MA, USA) was used to process and quantify the peak areas.

### 3.6. Mineral and Trace Elements Analysis

The determination of minerals and trace elements of the *C. melo* L. seeds was performed according to the method according to Albuquerque et al. (2013) [68]. Briefly, 0.5 mg of each sample was weighed into suitable Teflon digestion vessels. Nitric acid (4 mL), hydrogen peroxide (1 mL), and deionised water (3 mL) were carefully added, and the vials were tightly capped and placed in the microwave. For the digestion of the samples, a closed vessel microwave digestion system was used (Milestone ETHOS 1 Series, Milestone, Sorisole, Italy). After digestion, the digested samples were diluted with deionised water to 25 mL. Blank solutions were prepared to assess possible contaminations. The analysis of the minerals Ca, Mg, Na, K, and P, and the trace elements Fe, Zn, and Mn was carried out in an Inductively Coupled Plasma Optical Emission Spectrometer (ICP-OES Thermo iCAP serie 6000, Thermo Scientific Heraeus, Waltham, MA, USA).

### 3.7. Vitamin E Analysis

Vitamin E content of the *C. melo* L. pulp, peel, and seeds was determined according to the method reported by Alves et al. (2009) [69]. Briefly, 150 mg of each sample was weighed, and 75 µL of BHT solution (0.1%, *m*/*v*), 50 µL of tocol 0.1 mg/mL (internal standard), and 1 mL of absolute ethanol were added. The mixture was homogenised in an orbital vortex mixer for 30 min. Then, 2 mL of n-hexane were added, and the mixture was homogenised again for 30 min and left overnight at 4 °C. After a third homogenisation, 1 mL of NaCl (1%, *m*/*v*) was added. The mixture was vortexed and centrifuged for 2 min at 5000 rpm (Labofuge Ae centrifuge, Heraeus Sepatech, Hanau, Germany). After the separation of the organic phase, the residue was re-extracted twice with 2 mL of n-hexane. The organic phases were combined, and sodium sulphate anhydrous was added to the total extract. The mixture was vortexed and centrifuged for 5 min at 13,000 rpm (Heraeus Fresco 17 centrifuge, Thermo Fisher Scientific, Dreieich, Germany), and the organic phase was collected. The final extract was dried under a stream of nitrogen and re-suspended with 500 µL of n-hexane.

Separation and quantification were performed on an integrated HPLC system equipped with an MD-910 multi-wavelength DAD and an FP-920 fluorescence detector (Jasco, Tokyo, Japan), using an analytical column Supelcosil™ LC-SI (3 µm) 75 × 3.0 mm (Supelco, Bellefonte, PA, USA) at constant room temperature. The mobile phase was a mixture of n-hexane and 1,4-dioxane (98:2) eluted at 0.7 mL/min. The detection of compounds was performed by DAD connected in series to the fluorescence detector with excitation at 290 and emission at 330 nm. Quantification of compounds was performed based on the internal standard method using fluorescence signals. Chromatographic data were analysed using ChromNAV 2.0 HPLC Software from Jasco (Tokyo, Japan).

### 3.8. Total Vitamin C Analysis

The total vitamin C and L-ascorbic acid content of different parts of *C. melo* L. (pulp, peel, and seeds) was determined according to the method reported by Valente et al. (2014) [70]. A standard stock solution of L-ascorbic acid (1 mg/mL) was prepared on each day of analysis. The working solutions (1, 20, 40, 60, 80, and 100 µg/mL) were prepared by diluting the standard stock solution with mobile phase (20 mM ammonium dihydrogen phosphate, pH 3.5 (adjusted with orthophosphoric acid 85%, containing 0.015% (*w*/*v*) metaphosphoric acid). Briefly, 4 g of each sample were weighed, and 12 mL of acid solution, 10% (*v*/*v*) perchloric acid, and 1% (*w*/*v*) metaphosphoric acid in ultrapure water were added. The mixture was vortexed for 1 min. Then, the solution was diluted to 50 mL with mobile phase. The samples were filtered with a 150 mm diameter filter paper (Macherey-Nagel GmbH & Co. KG, Düren, Germany). Then, 1 mL of Tris [2-carboxyethyl] phosphine hydrochloride (5 mM) was added to 1 mL of the previously prepared solution and filtered. Separation and quantification were performed on an Alliance 2695 HPLC system (Waters, Milford, MA, USA), equipped with a Waters 2996 DAD, using a Synergi™ Hydro-RP analytical column (150 × 4.6 mm i.d., 4.0 µm particle size) with a SecurityGuard Cartridge AQ C18 (40 × 2.0 mm i.d., 5.0 µm particle size) from Phenomenex (Torrance, CA, USA). The total run time of the analysis was 6 min at a flow rate of 0.6 mL/min. The samples were monitored at 245 nm, and the injection volume was 20 µL. Column temperature was kept at 30 °C and the autosampler at 4 °C. The peak areas were processed and quantified with Empower™ software version 2.0 (Waters, Milford, MA, USA).

### 3.9. Antioxidant Activity and Total Phenolic Compounds

#### 3.9.1. Sample Extraction

For antioxidant activity evaluation and total phenolic compound determination, extracts of *C. melo* L. pulp and by-products were obtained. The seeds were first defatted with petroleum ether. Briefly, 3 g of the pulp, peel, and seeds were mixed with 30 mL of ethanol (90%, *v*/*v*) for 10 min at 4500 rpm and then filtered with a 150 mm diameter filter paper (Macherey-Nagel GmbH & Co. KG, Düren, Germany).

#### 3.9.2. Radical DPPH Scavenging Activity

For the determination of radical DPPH scavenging activity, 1 mL of ethanolic sample extract was mixed with 1 mL of DPPH ethanolic solution (0.004% *w*/*v*) [66]. After that, the mixture was left at room temperature, in the dark, and stirred for 1 h. Using a UV-Vis spectrophotometer (Thermo Scientific 300 Evolution, Madison, WI, USA), the absorbance of the sample extracts and the bleaching of DPPH were determined at 517 nm. As a standard, Trolox was utilised with concentrations ranging from 0.5 to 14 µg/mL. The results were expressed in mg of Trolox equivalents (TE) per 100 g of sample.

#### 3.9.3. Ferric Reducing Antioxidant Power Assay

Ferric-reducing antioxidant power was obtained according to the method described by Thaipong et al. (2006) [71]. Approximately 25 mL of acetate buffer (300 mM, pH 3.6), 2.5 mL of HCl solution (40 mM) of TPTZ (2,4,6-tripyridyl-s-triazine) (10 mM), and 2.5 mL of FeCl_3_.6H_2_O solution (20 mM) were mixed to prepare the working solution. This solution was incubated in a water bath at 37 °C for 10 min. Subsequently, 0.15 mL of the ethanolic sample extracts and 2.85 mL of the working solution were mixed in a glass tube and kept at room temperature, in the absence of light, for 30 min. Using a UV-Vis spectrophotometer (Thermo Scientific 300 Evolution, Madison, WI, USA), the absorbance of the samples was measured at 593 nm. The calibration curve was prepared with Trolox as a standard (2–105 µg/mL). The results were expressed in mg of TE per 100 g of sample.

### 3.10. Total Phenolics

The total phenolic content was determined according to the method reported by Song et al. (2010) [72], with some modifications. Briefly, 0.5 mL of ethanolic sample extracts were mixed with 2.5 mL of Folin–Ciocalteu reagent (0.2 M) for 4 min in the dark. Following this, 2.0 mL of sodium carbonate aqueous solution (7.5%, *w*/*v*) was added. The mixture was stirred and incubated in a water bath at 40 °C for 1 h. The absorbance of the samples was monitored in a UV/Vis spectrophotometer at 760 nm (Thermo Scientific Evolution 300, Madison, WI, USA). A calibration curve was prepared with gallic acid as a standard, with concentrations ranging from 2 to 90 µg/mL. The results were expressed in mg of gallic acid equivalents (GAE) per 100 g of sample.

### 3.11. Statistical Analysis

The statistical analyses of data were performed using Microsoft Office Excel^®^ 2016 and IBM^®^ SPSS Statistics 26.0 software. Results are expressed as mean and range. Values presented in the tables are the average values and range of three individual samples (*n* = 3) for each variety. For multiple comparisons of normally distributed data, parametric one-way analysis of variance (ANOVA) followed by the Tukey test was used, or the Student’s *t*-test was used. A value of *p* < 0.05 was considered statistically significant.

## 4. Conclusions

The by-products of *C. melo* L. have a highly interesting nutritional composition, offering a valuable source of nutrients that can enhance the nutritional quality of food products. Incorporating melon seeds and peels into food formulations can significantly increase their protein and dietary fibre contents, contributing to the fortification of food products with essential components for human health. Additionally, melon seeds present a remarkable amino acid profile, further increasing their nutritional value and relevance as a functional ingredient.

Both the peels and seeds contain important bioactive compounds, such as antioxidants and vitamins, which can promote health and help prevent diseases. The results of this study highlight the potential of melon by-products as valuable nutrient sources, with direct applicability in the food industry as functional ingredients. The use and valorisation of these by-products represent a promising alternative for the food industry and align with current consumer demands for healthier and more sustainable food options.

Moreover, using these by-products significantly contributes to food waste reduction and the efficient management of resources, positively impacting the circular economy. This work supports the transition towards more sustainable production practices in the food industry, helping to mitigate its economic, social, and environmental impact.

## Figures and Tables

**Table 1:** Nutritional composition and energy value (per 100 g dw) of different parts of *Cucumis melo* L. (mean value and range).

Components	Pulp		Peel		Seeds	
	Green Melon	Yellow Melon	Green Melon	Yellow Melon	Green Melon	Yellow Melon
Energy (kJ)	1374 (1358–1394) ^aA^	1371 (1355–1402) ^aA^	1022 (927–1081) ^aB^	957 (934–978) ^aB^	1707 (1556–1851) ^aC^	1744 (1553–1949) ^aC^
Energy (kcal)	324 (321–329) ^aA^	323 (320–330) ^aA^	245 (224–259) ^aB^	231 (226–235) ^aB^	414 (378–449) ^aC^	423 (378–472) ^aC^
Moisture (g)	8.89 (7.60–10.4) ^aA^	9.95 (8.49–11.5) ^aA^	8.51 (5.85–10.1) ^aA^	8.75 (7.80–10.6) ^aA^	7.97 (5.14–9.18) ^aA^	8.08 (6.03–12.0) ^aA^
Ash (g)	7.71 (4.52–9.32) ^aAB^	6.66 (6.42–6.97) ^aAC^	11.6 (9.64–14.9) ^aD^	11.0 (10.1–11.9) ^aBD^	3.62 (3.24–3.77) ^aC^	3.58 (3.31–3.88) ^aC^
Total protein (g) (NCF = 6.25)	8.21 (4.79–10.3) ^aA^	7.00 (5.86–8.31) ^aA^	10.2 (9.16–11.8) ^aA^	9.42 (8.41–10.1) ^aA^	21.3 (20.0–22.6) ^aB^	21.0 (19.6–23.0) ^aB^
Total fat (g)	0.653 (0.486–0.866) ^aA^	0.467 (0.357–0.662) ^aA^	1.09 (0.348–1.44) ^aA^	0.768 (0.506–1.05) ^aA^	27.5 (23.1–31.5) ^aB^	28.5 (22.9–34.9) ^aB^
Available Carbohydrates (g)	68.2 (63.5–71.7) ^aA^	69.7 (66.6–72.0) ^aA^	28.9 (18.4–35.0) ^aB^	23.0 (19.7–25.5) ^aB^	0.469 (0–1.48) ^aC^	1.31 (0–2.76) ^aC^
Total dietary fibre (g)	6.38 (3.38–8.98) ^aA^	6.21 (2.80–10.5) ^aA^	39.7 (32.4–51.4) ^aB^	47.1 (44.1–52.2) ^aB^	39.7 (35.7–44.2) ^aB^	39.0 (31.1–46.3) ^aB^

Values include several varieties. dw, dry weight; NCF, Nitrogen conversion factor. Mean values with ANOVA analysis—Different superscript lowercase letters are significantly different between the green and yellow melons for the different parts according to the Tukey test (*p* < 0.05). ANOVA analysis—Different superscript uppercase letters in the same row are significantly different according to the Tukey test (*p* < 0.05).

**Table 2:** Fatty acid composition (g/100 g dw) of green and yellow melon seeds (mean value and range).

Fatty Acid	Abbreviation	Green Melon Seeds	Yellow Melon Seeds
Myristic acid	C14:0	0.0106 (0.00859–0.0118) ^a^	0.0128 (0.0109–0.0157) ^a^
Pentadecanoic acid	C15:0	0.0076 (0.00476–0.00878) ^a^	0.0077 (0.00695–0.00824) ^a^
Palmitic acid	C16:0	2.48 (1.93–2.71) ^a^	2.45 (1.98–3.13) ^a^
Palmitoleic acid	C16:1c	0.0468 (0.0276–0.0682) ^a^	0.0526 (0.0475–0.0554) ^a^
Heptadecanoic acid	C17:0	0.0200 (0.0174–0.0217) ^a^	0.0222 (0.0188–0.0261) ^a^
*cis*-10-Heptadecanoic acid	C17:1c	0.0113 (0.00849–0.0128) ^a^	0.0093 (0.00671–0.0129) ^a^
Stearic acid	C18:0	1.33 (1.02–1.51) ^a^	1.43 (1.38–1.49) ^a^
Oleic acid	C18:1c	6.05 (3.21–7.53) ^a^	4.47 (3.66–5.49) ^a^
Linoleic acid	C18:2c (n6)	16.1 (14.0–19.2) ^a^	18.5 (13.9–24.7) ^a^
Linolenic acid	C18:3c (n3)	0.0541 (0.0443–0.0684) ^a^	0.0536 (0.0440–0.0722) ^a^
Arachidic acid	C20:0	0.0520 (0.0371–0.0577) ^a^	0.0479 (0.0458–0.0511) ^a^
*cis*-11-Eicosenoic acid	C20:1	0.0333 (0.0246–0.0393) ^a^	0.0327 (0.0267–0.0359) ^a^
Behenic acid	C22:0	0.0129 (0.0108–0.0139) ^a^	0.0102 (0.00914–0.0114) ^b^
Lignoceric acid	C24:0	0.0153 (0.0109–0.0181) ^a^	0.0149 (0.0108–0.0176) ^a^
Nervonic acid	C24:1	0.0144 (0.00348–0.0352) ^a^	0.0145 (0.00768–0.0263) ^a^
∑SFA		3.92 (3.05–4.33)	4.00 (3.47–4.74)
∑MUFA		6.16 (3.27–7.67)	4.58 (3.77–5.61)
∑PUFA		16.2 (14.1–19.2)	18.5 (14.0–24.7)

dw, dry weight. Values include several varieties. Different superscript letters within a row are significantly different according to Student’s *t*-test (*p* < 0.05). SFA—saturated fatty acids; MUFA—monounsaturated fatty acids; PUFA—polyunsaturated fatty acids.

**Table 3:** Amino acid composition (mg/100 g dw) of green and yellow melon seeds (mean value and range).

Amino Acid	Green Melon Seeds	Yellow Melon Seeds
Essential		
Histidine	147 (51.8–284) ^a^	247 (109–348) ^a^
Isoleucine	280 (174–399) ^a^	398 (296–495) ^a^
Leucine	967 (842–1254) ^a^	1034 (906–1104) ^a^
Lysine	144 (0–331) ^a^	301 (71.8–452) ^a^
Methionine	89 (0–273) ^a^	184 (0–294) ^a^
Phenylalanine	608 (500–730) ^a^	706 (638–809) ^a^
Threonine	296 (196–403) ^a^	403 (306–496) ^a^
Valine	443 (347–570) ^a^	521 (477–597) ^a^
Non-essential		
Alanine	762 (709–888) ^a^	798 (740–829) ^a^
Arginine	1673 (1439–2238) ^a^	1799 (1548–1957) ^a^
Aspartic acid	1896 (1480–2403) ^a^	1847 (1526–2186) ^a^
Cysteine	n.d.	n.d.
Glutamic acid	4327 (3191–5603) ^a^	4161 (3385–5269) ^a^
Glycine	996 (869–1266) ^a^	1026 (888–1103) ^a^
Proline	471 (368–578) ^a^	594 (532–669) ^a^
Serine	689 (585–856) ^a^	777 (703–866) ^a^
Tyrosine	303 (208–459) ^a^	423 (266–532) ^a^

dw, dry weight; n.d., not detected. Values include several varieties. Different superscript letters within a row are significantly different according to Student’s *t*-test (*p* < 0.05).

**Table 4:** Amino acid score according to the FAO requirement pattern for adults (mg/g protein) of green and yellow melon seeds (mean value and range).

Amino Acid	Requirement Pattern (mg/g Protein) ^1^	Amino Acid Score (%) ^2^	
		Green Melon Seeds	Yellow Melon Seeds
Histidine	15	45 (17–86) ^a^	78 (36–101) ^a^
Isoleucine	30	43 (28–61) ^a^	63 (49–72) ^a^
Leucine	59	77 (70–94) ^a^	84 (78–91) ^a^
Lysine	45	20 (10–33) ^a^	32 (8–44) ^a^
Threonine	23	60 (41–80) ^a^	83 (66–94) ^a^
Valine	39	53 (43–65) ^a^	64 (60–67) ^a^
Total sulfur amino acids (Met + Cys)	22	38 (19–56) ^a^	59 (58–60) ^a^
Total aromatic amino acids (Phe + Tyr)	38	112 (90–137) ^a^	141 (117–153) ^a^

^1^ FAO, 2013 [38]. ^2^ Amino acid score = (mg of amino acid in 1 g of test protein/mg of amino acid in requirement pattern) × 100. Met, Methionine; Cys, Cysteine; Phe, Phenylalanine; Tyr, Tyrosine. Values include several varieties. Different superscript letters within a row are significantly different according to Student’s *t*-test (*p* < 0.05).

**Table 5:** Mineral content (mg/100 g dw) of the green and yellow melon seeds (mean value and range).

Minerals	Green Melon Seeds	Yellow Melon Seeds
Phosphorus	624 (485–747) ^a^	669 (514–781) ^a^
Magnesium	292 (259–354) ^a^	299 (247–339) ^a^
Calcium	42.4 (28.2–60.8) ^a^	36.6 (30.5–45.0) ^a^
Potassium	805 (754–854) ^a^	799 (733–851) ^a^
Sodium	12.2 (7.77–19.8) ^a^	8.23 (6.71–9.63) ^a^
Manganese	1.96 (1.29–2.41) ^a^	2.12 (1.77–2.49) ^a^
Iron	5.49 (4.57–6.60) ^a^	5.81 (4.57–6.69) ^a^
Zinc	4.56 (3.56–5.97) ^a^	5.81 (4.55–7.20) ^a^

dw, dry weight. Values include several varieties. Different superscript letters within a row are significantly different according to Student’s *t*-test (*p* < 0.05).

**Table 6:** Vitamin E content (mg/100 g dw) of different parts of *Cucumis melo* L. (mean value and range).

	Pulp		Peel		Seeds	
	Green Melon	Yellow Melon	Green Melon	Yellow Melon	Green Melon	Yellow Melon
α-tocopherol	0.43 (0.084–0.80) ^aA^	0.37 (0.11–0.57) ^aA^	4.0 (1.2–6.2) ^aB^	1.3 (0.99–1.5) ^aAB^	1.6 (1.3–1.9) ^aAB^	2.2 (1.1–4.0) ^aAB^
β-tocopherol	0.028 (0.018–0.036) ^aA^	0.021 (0.019–0.024) ^aA^	0.13 (0.048–0.19) ^aB^	0.051 (0.042–0.057) ^aA^	n.d.	n.d.
γ-tocopherol	0.14 (0.032–0.32) ^aA^	0.17 (0.092–0.24) ^aA^	13 (6.8–19) ^aC^	2.9 (1.9–3.7) ^bA^	5.8 (4.7–7.0) ^aAB^	12 (8.8–14) ^aBC^
γ-tocotrienol	0.10 (0.036–0.17) ^aA^	0.10 (0.088–0.12) ^aA^	0.73 (0.65–0.92) ^aB^	0.73 (0.52–0.87) ^aB^	0.39 (0.20–0.60) ^aAB^	0.32 (0.20–0.51) ^aA^
δ-tocopherol	0.033 (0.024–0.040) ^aA^	0.029 (0.026–0.035) ^aA^	2.3 (1.7–2.9) ^aB^	0.25 (0.16–0.33) ^bA^	0.27 (0.16–0.40) ^aA^	0.31 (0.18–0.43) ^aA^

dw, dry weight; n.d., not detected. Values include several varieties. ANOVA analysis—Different superscript lowercase letters are significantly different between the green and yellow melons for the different parts according to the Tukey test (*p* < 0.05). ANOVA analysis—Different superscript uppercase letters in the same row are significantly different according to the Tukey test (*p* < 0.05).

**Table 7:** DPPH radical scavenging activity, FRAP assay, total phenolics, and total vitamin C contents of different parts of *Cucumis melo* L. (mean value and range).

	Pulp		Peel		Seeds	
	Green Melon	Yellow Melon	Green Melon	Yellow Melon	Green Melon	Yellow Melon
DPPH (mg TE/100 g dw)	88.1 (9.98–204) ^aA^	52.3 (8.99–135) ^aA^	144 (25.3–281) ^aA^	120 (25.1–305) ^aA^	4.59 (3.91–5.39) ^aA^	5.57 (2.88–8.35) ^aA^
FRAP (mg TE/100 g dw)	2919 (288–7216) ^aA^	1851 (283–4943) ^aA^	7408 (2586–13,597) ^aA^	6146 (1361–15,504) ^aA^	3338 (2803–4131) ^aA^	3269 (1557–4953) ^aA^
Total phenolics (mg GAE/100 g dw)	1150 (74.2–2208) ^aA^	604 (86.8–1614) ^aA^	2212 (693–3887) ^aA^	1976 (466–4911) ^aA^	201 (77.1–288) ^aA^	195 (18.7–340) ^aA^
Total vitamin C (mg/100 g dw)	56.7 (0–116) ^aA^	26.2 (0–78.7) ^aA^	70.3 (0–182) ^aA^	31.2 (0–93.5) ^aA^	n.d.	n.d.

Values include several varieties. dw, dry weight; n.d., not detected; TE, Trolox equivalents; GAE, gallic acid equivalents. ANOVA analysis—Different superscript lowercase letters are significantly different between the green and yellow melons for the different parts; according to the Tukey test (*p* < 0.05). ANOVA analysis—Different superscript uppercase letters in the same row are significantly different; according to the Tukey test (*p* < 0.05).

## Data Availability

The original contributions presented in this study are included in the article. Further enquiries can be directed to the corresponding author.
